# Effectiveness of organizational interventions vs usual care for reducing incidence and mitigating impact of workplace violence on registered nurses: a systematic review protocol

**DOI:** 10.11124/JBIES-25-00371

**Published:** 2026-05-18

**Authors:** Domenico Cannizzaro, Ilaria Saguatti, Dalia Caleffi, Sergio Rovesti, Paola Ferri

**Affiliations:** 1 Clinical and Experimental Medicine Ph.D. Program, Department of Biomedical, Metabolic and Neural Sciences, University of Modena and Reggio Emilia, Modena, Italy; 2 Department of Biomedical, Metabolic and Neural Sciences, University of Modena and Reggio Emilia, Modena, Italy

**Keywords:** intervention strategies, organizational interventions, registered nurses, workplace violence

## Abstract

**Objective:**

The objective of this review will be to evaluate the effectiveness of multilevel interventions, including communication strategies and other organizational measures targeting structures, processes, policies, and culture, compared to usual care or no intervention, in reducing the incidence and severity of verbal and physical workplace violence against registered nurses.

**Introduction:**

Workplace violence against nurses includes verbal, physical, and psychological abuse, with lasting consequences on their mental health, job satisfaction, and workforce retention. Although awareness of the phenomenon has increased, the existing evidence on prevention and management interventions remains fragmented and inconclusive. This review will synthesize the current evidence to identify effective strategies, thereby supporting institutional decision-making and protecting frontline nurses.

**Eligibility criteria:**

This review will consider experimental, quasi-experimental, and observational studies that evaluate interventions aimed at preventing, managing, or mitigating workplace violence against registered nurses.

**Methods:**

The proposed review will follow the JBI methodology for systematic reviews of effectiveness. The main databases to be searched will include MEDLINE Ultimate (EBSCOhost), Embase (Embase.com), CINAHL (EBSCOhost), PsycINFO (EBSCOhost), Scopus, Web of Science Core Collection, Cochrane CENTRAL, ProQuest Dissertations and Theses Global, and Google Scholar (first 200 records). Additional records will be identified through forward and backward citation tracking. Two reviewers will independently review the studies, assess methodological quality, and extract data. Data synthesis will be performed and the certainty of the evidence will be assessed using the Grading of Recommendations, Assessment, Development and Evaluation (GRADE) approach. No date restrictions will be applied (from database inception to January 9, 2026). Studies published in English will be included, while studies in other languages will be considered with translation support at the title and abstract level.

**Review registration:**

PROSPERO CRD420251125105

## Introduction

Workplace violence (WPV) against registered nurses (RNs) is a growing public health concern that poses serious challenges to the provision of safe and effective health care services.^1^ WPV is increasingly recognized as a widespread problem affecting health care systems globally.^2^ The European Agency for Safety and Health at Work (EU-OSHA) defines WPV as “the intentional use of force, threatened or actual, against another person or group, in work-related circumstances, which causes or is likely to cause injury, death, psychological harm, developmental harm, or deprivation.”^3^^(para.2.2)^ Incidents of WPV mainly involve verbal threats and, to a lesser extent, theft, damage to property, or physical assault.^4^

WPV has been linked to several negative outcomes, including lower job satisfaction, reduced organizational commitment, emotional demoralization, burnout, psychological stress, and increased staff turnover.^5^ These incidents diminish RNs’ sense of safety and raise critical concerns about the nurse–patient relationship. If left unaddressed, WPV can undermine the credibility and perceived safety of health care institutions.^6^,^7^

A cross-sectional study conducted in Germany on 349 health care workers found that 87% of participants had experienced physical violence from patients and 64% from family members of patients.^8^ A survey conducted among 539 nurses in an Italian hospital revealed that the most common type of WPV was verbal, particularly in general hospitals (64%) and emergency departments (54%).^9^ Men committed acts of physical violence significantly more often than women (χ^2^ test, *P*=0.034). Verbal violence (51%) was often committed by people in a lucid and normal state of consciousness.^10^

Nurses exposed to repeated episodes of violence in the workplace may suffer long-term consequences, including psychological distress and professional disengagement, thereby compromising the quality of care.^5^,^11^ Empirical evidence indicates that the psychological and professional repercussions of WPV can persist for months or even years.^11^

The COVID-19 pandemic has added considerable stress to health care settings, contributing to increased incidents of WPV.^12^ A recent review highlighted that aggression toward nurses has harmful effects, underscoring the need for institutional protection policies.^13^ A study conducted in Australia reported that over 75% of nurses and midwives had experienced violence from patients and visitors in the previous 6 months.^14^ Sulistyowati *et al.* examined the post-COVID-19 relationship between WPV and job satisfaction among nurses in an Indonesian public hospital. Despite the reduction in COVID-19 cases, RNs continued to face excessive workloads and remained vulnerable to WPV.^15^ Somani *et al.* evaluated various interventions to reduce the incidence and severity of WPV against RNs.^16^ Although structured training programs have shown some positive impact, their overall effectiveness appeared to be limited.

Recent technological advances have led to the development of various digital tools, including online training platforms, real-time alert systems, mobile applications, wearable devices, and artificial intelligence–based solutions to improve the prevention and management of WPV. However, the evidence on the effectiveness of these interventions remains fragmented and heterogeneous, and no comprehensive systematic reviews have consistently synthesized the available data.^17^ Within this evolving landscape, artificial intelligence has been increasingly investigated not only as a supportive tool but also as a potential proactive prevention approach, further underscoring the rapid development of the field and the need for structured evidence synthesis.^18^

This systematic review aims to identify and evaluate organizational strategies, protocols, and policies for preventing or mitigating WPV against RNs. It will examine all forms of WPV and assess the effectiveness of current prevention and management approaches. The goal is to determine which interventions best reduce WPV and support affected nurses. The review also seeks to fill a critical gap in the literature by rigorously evaluating the effectiveness of existing strategies, policies, and protocols aimed at managing, preventing, or eliminating verbal and physical violence against RNs.

A preliminary search was conducted of PROSPERO, PubMed, the Cochrane Database of Systematic Reviews, and *JBI Evidence Synthesis*. While several systematic reviews addressing aspects of WPV interventions were identified, these reviews differ in scope, target populations, and methodological approaches, and none has specifically examined organizational and digital interventions targeting RNs through an integrated synthesis.^19^,^20^

The present analysis will provide an evidence-based synthesis of interventions to guide health policies, inform institutional safety strategies, and enhance nurses’ physical and psychological well-being, ultimately contributing to improved health care quality.

## Review question

What is the effectiveness of multilevel organizational interventions, including communication strategies, structured reporting systems, environmental risk assessments, early warning protocols, and operational policies, compared with usual care or no structured intervention, in reducing the incidence and severity of verbal and physical WPV against RNs?

## Eligibility criteria

The eligibility criteria for this review were developed based on the PICO framework, which takes into account 4 key elements: population, intervention, comparator, and outcome^21^ (see Appendix I).

### Population

This review will consider studies that include RNs, regardless of demographics or health care setting. Studies involving mixed samples of health care professionals will be eligible if RN-specific data are reported separately or can be reliably extracted from subgroup analyses, tables, or supplementary materials. Studies including mixed nursing populations where RN-specific data cannot be fully isolated will still be considered eligible if RNs represent the majority of the sample. Studies in which RN-specific data cannot be reliably isolated or where RNs do not constitute the predominant professional group will be excluded.

### Interventions

This review will consider studies that evaluate organizational interventions aimed at preventing and managing WPV against RNs. These interventions may be single-component or multi-faceted and may include communication protocols, structured incident reporting systems, environmental risk assessments, early warning protocols, and the implementation of operational policies. Interventions may vary in terms of intensity, mode of delivery, and duration, as defined by the individual studies.

### Comparators

This review will consider studies comparing the intervention with passive comparators, such as no intervention or usual institutional practices, or with active comparators, including alternative strategies or variations of the same intervention.

In this review, *usual care/support* will be defined as routine, non-structured practices provided by health care organizations that are not intentionally designed, formally implemented, or evaluated as WPV prevention or management interventions. Pre-existing policies or procedures are considered usual care only when they are not part of a formal, multi-component, or explicitly preventive organizational program.

Where applicable, alternative interventions focused on WPV implemented as separate arms in comparative or multi-arm studies will also be considered. Comparator characteristics, such as mode of administration (passive or interactive), frequency, intensity, setting, and timing, will be described when available.

### Outcomes

This review will consider studies that include primary, secondary, and tertiary outcomes related to the prevention and management of WPV against RNs. The severity of verbal and physical WPV will be operationalized according to the definitions and measurement approaches adopted in the primary studies, including indicators such as intensity of verbal aggression, presence and seriousness of physical injury, need for medical treatment, or use of graded severity scales. The severity of the violence will be synthesized quantitatively or narratively depending on data comparability.

The primary outcome of interest will be the frequency of WPV, defined as the occurrence of verbal and/or physical violent episodes over a specified observation period. Frequency may be measured using rates of violent incidents, proportions of nurses experiencing at least one violent episode during the observation period, or absolute counts of reported incidents, as reported by the included studies. A hierarchy of measurement sources will be applied, prioritizing structured institutional incident reporting systems, followed by validated self-administered questionnaires, and, when no other sources are available, non-validated self-reported or anecdotal data. Outcomes will be extracted at all reported time points and categorized as short term (<6 months), medium term (6–12 months), and long term (>12 months). When multiple measures of the same outcome are reported within a study, all available measures will be extracted; the measure selected for primary analysis will be based on the highest-ranked measurement source, the longest available follow-up, and comparability across studies.

Secondary outcomes will include indicators of personal well-being and safety, such as psychological distress, perceptions of workplace safety, burnout symptoms, and self-efficacy in managing violent incidents, measured using validated scales or self-administered questionnaires.

Tertiary outcomes will include organizational and occupational indicators, such as absenteeism, turnover, and changes in employment patterns (eg, transition to part-time work or departmental transfer), derived from administrative data or organizational reports and reflecting broader institutional impact. Secondary and tertiary outcomes will be extracted for all reported time points and categorized as short term (<6 months), medium term (6–12 months), or long term (>12 months).

### Types of studies

This review will consider both experimental and quasi-experimental study designs, including randomized controlled trials, non-randomized controlled trials, before-and-after studies, and interrupted time-series studies. In addition, analytical observational studies, including prospective and retrospective cohort studies, case-control studies, and analytical cross-sectional studies, will be considered for inclusion.

The inclusion of analytical observational studies is justified by the organizational nature of the interventions; their findings will be interpreted cautiously through formal risk-of-bias assessment and will be considered supportive rather than causal.

This review will also consider descriptive observational study designs such as case series, individual case reports, and descriptive cross-sectional studies. Studies assessing digital or technological interventions (as described previously), including mobile health applications, artificial intelligence–driven monitoring tools, or virtual reality platforms, will be considered if specifically designed to address WPV among RNs. However, editorials, commentaries, conference abstracts, methodological articles, and literature reviews will not be considered in this review.

## Methods

The proposed systematic review will be conducted in accordance with the JBI methodology for systematic reviews of effectiveness^22^ and reported in line with the Preferred Reporting Items for Systematic Reviews and Meta-Analyses (PRISMA) guidelines.^23^ The protocol is registered in PROSPERO (CRD420251125105).

### Search strategy

The search strategy will aim to locate both published and unpublished studies. An initial limited search of MEDLINE Ultimate (EBSCOhost) and CINAHL (EBSCOhost) was undertaken to identify articles on the topic. The text words contained in the titles and abstracts of relevant articles, and the index terms used to describe the articles, were used to develop the full and reproducible search strategy for MEDLINE Ultimate (EBSCOhost), which is reported in Appendix II. The MEDLINE strategy will be adapted for each included information source, and the complete search strategies for all databases will be provided in the final protocol. The reference lists of all studies selected for critical appraisal will be screened for additional studies.

Studies published in English will be included. Studies in other languages will also be considered, with translation support for title and abstract screening. Incomplete translation resources may lead to residual language bias and potentially limit comprehensiveness of the evidence base. Studies published from database inception to January 9, 2026, will be considered, with no date restrictions.

The databases to be searched will include MEDLINE Ultimate (EBSCOhost), Embase (Embase.com), CINAHL (EBSCOhost), PsycINFO (EBSCOhost), Scopus, Web of Science Core Collection, Cochrane CENTRAL, ProQuest Dissertations and Theses Global, and Google Scholar. Sources of unpublished studies and gray literature will be identified through manual checking of the references of the included studies. For Google Scholar and ProQuest Dissertations and Theses Global, the first 200 results per query will be screened, with full texts retrieved only for relevant empirical reports. Gray literature will be searched to identify potentially relevant studies and minimize publication bias; only studies meeting the eligibility criteria and reporting empirical data on interventions will be considered for synthesis of effectiveness outcomes.

An expert research librarian assisted with refining the search strategy. The final strategy will be adapted for each selected database using Systematic Review Accelerator (SRA; Bond University, Robina, Australia). An expert health sciences librarian will also conduct an internal peer review using the Peer Review of Electronic Search Strategies (PRESS) 2022 checklist to ensure methodological rigor of the strategy.^24^

The search strategy was originally developed and piloted in July 2025 and was subsequently rerun and finalized on January 9, 2026, following peer-review feedback to ensure completeness and reproducibility.

### Study selection

Following the search, all identified citations will be collated and uploaded into EndNote v.21 (Clarivate Analytics, PA, USA), and duplicates removed. Prior to full screening, a pilot test will be conducted on a random sample of approximately 50 titles and abstracts by 2 independent reviewers to ensure consistent application of the eligibility criteria. Inter-reviewer agreement of at least 80% will be considered acceptable before proceeding to the full-text screening phase. Discrepancies will be discussed and, if necessary, the eligibility criteria will be refined. A second pilot round will be conducted if agreement is below the predefined threshold.

Titles and abstracts will then be independently screened by 2 reviewers against the eligibility criteria. The deduplicated records will be exported to Rayyan QCRI (Qatar Computing Research Institute [Data Analytics], Doha, Qatar), a free web-based software designed to facilitate systematic reviews and support collaborative screening. Once satisfactory agreement between the reviewers has been confirmed, they will independently screen the full set of identified articles. Potentially relevant studies will be retrieved in full, and their citation details imported into JBI System for the Unified Management, Assessment and Review of Information (JBI SUMARI; JBI, Adelaide, Australia).^25^ The full text of selected citations will be independently evaluated in detail against the eligibility criteria by 2 reviewers. Reasons for exclusion of full-text studies that do not meet the eligibility criteria will be systematically documented and reported in the final review. Any disagreements that arise between the reviewers at each stage of the study selection process will be resolved through discussion or, if necessary, in consultation with a third reviewer. The results of the search and study selection process will be reported in full in the final systematic review and presented in a PRISMA flow diagram.^23^ The reasons for exclusion will be tabulated and attached as an appendix in the final review. If any eligible studies are authored by members of the review team, these studies will be screened and appraised independently by reviewers not involved in the study.

### Assessment of methodological quality

Eligible studies will be critically appraised by 2 independent reviewers at the study level for methodological quality in the review using standardized critical appraisal instruments from JBI for experimental, quasi-experimental, and observational studies.^26^-^28^ Appropriate checklists will be selected according to the study design (eg, randomized controlled trials, quasi-experimental studies, or observational designs).

Prior to the full critical appraisal process, a pilot critical appraisal will be performed independently by 2 reviewers on 3 studies to assess consistency in the interpretation and application of the JBI appraisal tools. Agreement of at least 80% will be required before proceeding to the full appraisal. Any discrepancies will be resolved through discussion or consultation with a third reviewer, and clarification notes will be added to the appraisal protocol if needed. Authors of papers will be contacted to request missing or additional data for clarification, where required. The results of critical appraisal will be reported in a table with accompanying narrative.

All eligible studies will be included in the synthesis. The critical appraisal findings will inform interpretation and confidence in effect estimates. Studies at high risk of bias will be interpreted cautiously and examined through sensitivity analyses. Differences between analyses will be reported when assessing result robustness. For cluster randomized trials, additional sources of bias such as recruitment bias, baseline imbalance, loss of clusters, and incorrect unit-of-analysis will be specifically considered during critical appraisal, in line with Cochrane methodological guidance.^29^

### Data extraction

Data will be independently extracted by 2 reviewers using the standardized JBI data extraction tool, JBI SUMARI,^25^ adapted to the study design. The extraction process will be adapted to the study design to ensure that the methodological and outcome data are comprehensively captured. If any modifications are made to the original tool, these will be described in detail and attached to the manuscript. The extracted information will include specific details about the study population, methodological characteristics, type of intervention (including scope, origin, implementation process, and personnel involved), relevant outcomes, and key findings relevant to the objectives of the review.

Prior to full data extraction, pilot data extraction will be undertaken independently by 2 reviewers on 3 included studies using the standardized JBI data extraction tool. The extracted data will be compared to assess consistency in coding of population characteristics, interventions, comparators, and outcomes. An agreement threshold of at least 80% will be considered acceptable. Where discrepancies are identified, the extraction form will be refined, and a second pilot test will be conducted, if necessary. Any disagreements that arise between the reviewers will be resolved through discussion or with a third reviewer. Study authors will be contacted to obtain missing data, where required.

### Data synthesis

Studies will, where possible, be pooled with statistical meta-analysis using JBI SUMARI.^25^ All analyses will be conducted within a single synthesis framework aligned with the review question. Studies with passive and active comparators will be synthesized separately, with active comparisons analyzed narratively. Separate meta-analyses will be performed for distinct intervention types or outcome domains, if identified. Effect sizes will be expressed as either odds ratios (for dichotomous data) or weighted (or standardized) final post-intervention mean differences (for continuous data) and their 95% CIs will be calculated for analysis. Cluster randomized trials will be identified and analyzed using appropriate statistical methods. Where clustering effects have not been accounted for in the original analyses, effective sample size adjustments will be applied using reported intraclass correlation coefficients (ICCs) or, when unavailable, externally derived ICC estimates, in accordance with the recommendations of the Cochrane Handbook for Systematic Reviews of Interventions.^29^ If adjustment is not feasible due to insufficient data, the results will be synthesized narratively and their limitations will be explicitly reported. For the primary outcome, effect measures will be selected according to reported measurement units and pooled only when outcomes are comparable.

Heterogeneity will be assessed statistically using the standard χ^2^ and *I*^2^ tests. An *I*^2^ value of 0–40% will be interpreted as low heterogeneity, 30–60% as moderate, 50–90% as substantial, and 75–100% as considerable, in accordance with the thresholds recommended by the *JBI Manual for Evidence Synthesis*.^30^ Statistical analyses will be performed using a random effects model, in accordance with the decision‑making framework recommended by Tufanaru *et al.*^22^ If data are missing or reported ambiguously, study authors will be contacted via email. If no response is received, predefined and methodologically appropriate imputations will be applied and examined through sensitivity analyses. Conversions between effect measures (eg, odds ratios to risk ratios) will be undertaken only when required to ensure metric comparability. All imputations and data transformations will be explicitly reported and examined through sensitivity analyses.

Subgroup analyses will be conducted where data permit. Sensitivity analyses will be conducted to test decisions made regarding the robustness of the results by verifying the impact of methodological decisions, such as the inclusion of studies at high risk of bias or the use of imputed data. Where statistical pooling is not possible, the findings will be presented in narrative format, including tables and figures to aid in data presentation, where appropriate. A structured narrative synthesis will be conducted following JBI guidelines, combining tabular summaries with descriptive text organized by intervention type and outcome domain, and supported by summary tables and figures. All synthesis methods will be conducted in accordance with the current guidelines of the *JBI Manual for Evidence Synthesis.*^30^

### Assessing certainty in the findings

The Grading of Recommendations, Assessment, Development and Evaluation (GRADE) approach for grading the certainty of evidence will be followed^31^ and a Summary of Findings (SoF) will be created using GRADEpro GDT software, (McMaster University, ON, Canada). The SoF will present the following information where appropriate: absolute risks for the intervention and comparator groups for dichotomous outcomes; estimates of relative or absolute effect sizes (eg, risk ratios, incidence rate ratios, mean differences/standardized mean differences, as applicable); and a rating of the overall certainty of the evidence based on the domains of risk of bias, directness, heterogeneity, precision, and risk of publication bias. The SoF will be limited to the primary outcomes of incidence/frequency and severity of verbal and physical WPV. Secondary psychological and organizational outcomes will be synthesized narratively.

## Acknowledgments

Carmela Palazzi, head of the Medical Library at the University of Modena and Reggio Emilia, for her valuable assistance in developing the search strategy.

## Author contributions

PF, DC, and SR conceived and designed the study (conceptualization, methodology). DC drafted the initial version of the protocol and developed the literature search strategy, with support from an experienced information specialist (writing of the original draft, investigation). DC, IS, and DaC will conduct screening, study selection, and data extraction (data curation). DC and PF contributed to the critical revision and editing of the manuscript (review and editing). PF and SR supervised the project design and will continue to oversee its implementation. All authors reviewed and approved the final version of this protocol for submission.

## Appendix I: Eligibility criteria using the PICO framework

**Table:**

	Inclusion criteria	Exclusion criteria
**Population**	RNs (of any gender or age) working in hospitals or community clinical settings, regardless of any previous exposure to WPV	Studies involving nursing students or other health care professionals without the possibility of extracting specific data relating to RNs
**Intervention**	Interventions, including organizational strategies, communication strategies, institutional policies, operational protocols, and integrated training or technology-based components, targeting organizational structures, processes, and culture, aimed at preventing, managing, or reducing WPV against RNs, in both the pre-incident and post-incident phases	Studies on interventions aimed at reducing secondary outcomes resulting from WPV
**Comparison**	No intervention, usual care, or alternative active strategies implemented in comparative or multi-arm designs	None
**Outcome**	**Primary outcome:** Frequency of WPV episodes (verbal or physical). **Secondary outcome:** Knowledge, attitudes, and skills related to WPV prevention and de-escalation; psychological well-being (eg, distress, burnout, self-efficacy); perceived safety; adverse personal and organizational consequences	Studies that do not allow separate analysis of data specific to RNs or exclusively assess secondary outcomes unrelated to WPV management or prevention

RN, registered nurse; WPV, workplace violence.

## Appendix II: Search strategy

**Table:**

MEDLINE Ultimate (EBSCOhost)		
Search conducted: January 9, 2026		
Search	Query	Records retrieved
#1	MH “Nurses”	49,793
#2	((Nurs* OR “Registered Nurse*” OR Nurse) N3 (Nursing OR “Nurse Clinician*” OR “Nurse Practitioner*”)) TI OR AB	427,958
#3	(“Nursing staff” OR “Nursing staff hospital” OR “Healthcare workers” OR “Nursing staff hospital” OR “Nurs* staff”) TI OR AB	427,957
#4	#1 OR #2 OR #3	477,494
#5	MH “Workplace Violence” OR MH “Violence” OR MH “Physical Abuse” OR MH “Aggression”	74,512
#6	(violence OR aggress* OR fight* OR abuse* OR harass* OR threat* OR incivil* OR assault*) TI OR AB	427,992
#7	#5 OR #6	501,993
#8	MH “Intervention Studies”	94,987
#9	(policy OR plan OR guideline* OR strateg* OR protocol* OR program* OR intervention* OR management) TI OR AB	428,893
#10	#8 OR #9	448,235
#11	#4 AND #7 AND #10	427,956
